# Defects in Tendon, Ligament, and Enthesis in Response to Genetic Alterations in Key Proteoglycans and Glycoproteins: A Review

**DOI:** 10.1155/2013/154812

**Published:** 2013-11-10

**Authors:** Subhash C. Juneja, Christian Veillette

**Affiliations:** Arthritis Program, Division of Orthopaedic Surgery, Toronto Western Hospital, Toronto, ON, Canada M5T 2S8

## Abstract

This review summarizes the genetic alterations and knockdown approaches published in the literature to assess the role of key proteoglycans and glycoproteins in the structural development, function, and repair of tendon, ligament, and enthesis. The information was collected from (i) genetically altered mice, (ii) *in vitro *knockdown studies, (iii) genetic variants predisposition to injury, and (iv) human genetic diseases. The genes reviewed are for small leucine-rich proteoglycans (lumican, fibromodulin, biglycan, decorin, and asporin); dermatan sulfate epimerase (Dse) that alters structure of glycosaminoglycan and hence the function of small leucine-rich proteoglycans by converting glucuronic to iduronic acid; matricellular proteins (thrombospondin 2, secreted phosphoprotein 1 (Spp1), secreted protein acidic and rich in cysteine (Sparc), periostin, and tenascin X) including human tenascin C variants; and others, such as tenomodulin, leukocyte cell derived chemotaxin 1 (chondromodulin-I, ChM-I), CD44 antigen (Cd44), lubricin (Prg4), and aggrecan degrading gene, a disintegrin-like and metallopeptidase (reprolysin type) with thrombospondin type 1 motif, 5 (*Adamts5*). Understanding these genes represents drug targets for disrupting pathological mechanisms that lead to tendinopathy, ligamentopathy, enthesopathy, enthesitis and tendon/ligament injury, that is, osteoarthritis and ankylosing spondylitis.

## 1. Introduction 

### 1.1. Proteoglycans and Glycoproteins

Proteoglycans are proteins that are heavily glycosylated. The basic proteoglycan unit consists of a core protein with one or more covalently attached GAG chain(s) at specific site(s) [1]. The GAG chains comprise disaccharide units composed of aminoglycan and uronic acid (glucuronic and or iduronic acid) [2]. The chains are long, linear carbohydrate polymers that are negatively charged under physiological conditions, due to the occurrence of sulfate and uronic acid groups. Proteoglycans occur in the connective tissue. Glycoproteins, on the other hand, are proteins that contain oligosaccharide/glycans attached to polypeptide side chains covalently. The carbohydrate is attached to the protein in a cotranslational or posttranslational modification via glycosylation. Secreted extracellular proteins are often glycosylated [3]. In the proteins that have extracellular segments, those segments are also glycosylated. Glycoproteins are often important integral membrane proteins, where they play a role in cell-cell interactions.

### 1.2. Tendon, Ligament, and Enthesis

**Figure 1:**
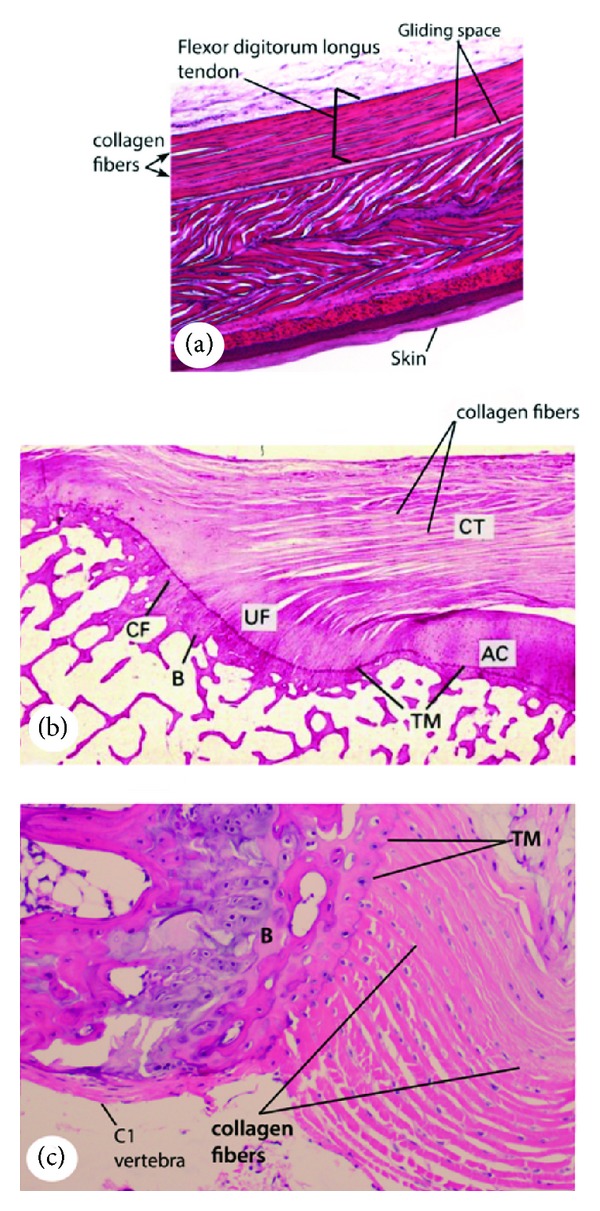
(a) Longitudinal section of FDL tendon from a 3-month old mouse. Tendon is composed of parallel collagen fibers and cells aligned (dark blue) in the direction of fibers. Gliding space is shown. (b) The normal structure of fibrocartilaginous entheses. The 4 zones of tissue at the insertion of the human supraspinatus tendon: dense fibrous connective tissue (CT), uncalcified fibrocartilage (UF), calcified fibrocartilage (CF), and bone (B). Reprinted from Benjamin and Ralphs (1998) [13], 1998, Anatomical Society of Great Britain and Ireland, with permission from John Wiley and Sons. AC, articular cartilage; TM, tidemark. (c) Annulus fibrosus fibers between tail vertebra C1 (shown) and C2 (not shown) from 3-month old mouse, showing entheses.

**Figure 2:**
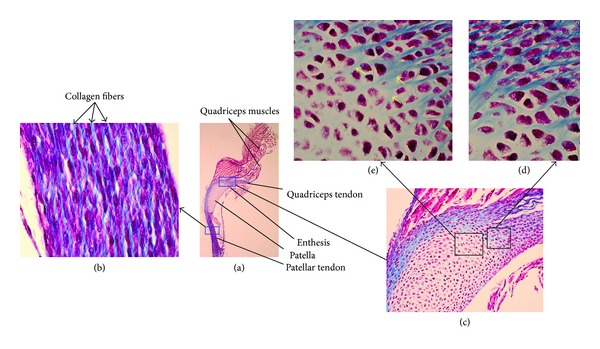
(a) Masson's trichrome staining of postnatal day 1 patella with patellar and quadriceps tendon in mouse at 4x. (b) Higher magnification (100x) shows patellar tendon composed of parallel collagen fibers (blue) with cells aligned almost parallel to fibers. Enthesis in rectangle (i.e., quadriceps tendon insertion on patella) is shown (a). Higher magnification of enthesis at 20x (c) and at 100x ((d) and (e)). Collagen fibers (blue) show penetration on patella (d) and ending on patella ((e), yellow arrows).

A tendon is a compositionally complex tissue with a predominantly mechanical function: translating muscular contractions into joint movement by transmitting forces from muscle to bone [4]. Histologically, tendons consist of dense regular connective tissue fascicles encased in dense irregular connective tissue sheaths. Tendons are composed mostly of parallel arrays of collagen fibers closely packed together (Figures 1(a), 1(b), and 2(b)). The dry mass of normal tendons, which makes up about 30% of the total mass with water, is composed of about 86% collagen, 2% elastin, 1%–5% proteoglycans, and 0.2% inorganic components such as copper, manganese, and calcium [5]. The collagen portion is made up of 97%-98% Col I, with small amounts of other types of collagen [5, 6]. The tenocytes produce the collagen molecules, which aggregate end-to-end and side-to-side to produce collagen fibrils. Fibril bundles are organized to form fibers with the elongated tenocytes closely packed between them. There is a three-dimensional network of cell processes associated with collagen in the tendon. The cells communicate with each other through gap junctions, and this signaling gives them the ability to detect and respond to mechanical loading [7]. A ligament is the fibrous tissue similar to tendon except that it connects bone to other bone. Periodontal ligament comprises group of fibers that attach the cementum of teeth to the surrounding alveolar bone.

Enthesis (plural, entheses) is the point at which a tendon or ligament or muscle inserts into bone, where the collagen fibers are mineralized and integrated into bone tissue. There are two types: fibrous entheses and fibrocartilaginous entheses. In a fibrous enthesis, the collagenous tendon or ligament directly attaches to the bone, whilst the fibrocartilaginous enthesis displays 4 zones during the transition from tendon/ligament to bone: (i) tendon area displaying longitudinally oriented fibroblasts and a parallel arrangement of collagen fibers, (ii) a fibrocartilaginous region of variable thickness where the structure of the cells changes to chondrocytes, (iii) an abrupt transition from cartilaginous to calcified fibrocartilage—the so-called tidemark, and (iv) bone [8]. 

Tendon or ligament and bone display dramatically different mechanical behavior [9, 10]. At the hierarchical level of the tissue, tendon has a tensile modulus on the order of 200 MPa in the direction of muscle force, but buckles in compression (i.e., it behaves like a rope) [10]. Bone, on the other hand, has a modulus of 20 GPa in both tension and compression, and is rigid and brittle relative to tendon [9]. The attachment of a compliant material like tendon to a relatively stiff material like bone is a fundamental engineering challenge [11, 12]. Enthesis is a common region for overuse injuries. It is targeted in ankylosing spondylitis and psoriatic arthritis [8, 13]. A few images of enthesis are shown in Figures 1(b), 1(c), and 2(c).

### 1.3. Genetics and Knockdown Approaches

Gene knockout mice [14], *in vitro* knockdown studies [15], gene overexpressing mice [15], patients with genetic disorder [16], and subjects with genetic variants linked to susceptibility to disease [17] are useful tools to better understand the gene functions and human diseases and to develop therapeutical strategies to treat the human diseases. Tendons, ligaments, and entheses are the key elements of musculoskeletal function. A number of genes for proteoglycans and glycoproteins play role in their development, structure, and function. This review summarizes the up-to-date published literature on the role of key proteoglycans and glycoproteins which affects the structural development, function, mechanical and viscoelastic properties, healing process, and calcification in tendons, ligaments and entheses at young age, adulthood, and or during aging. The information has been collected from (i) mice deleted in single or double genes, (ii) mice presenting different content of proteoglycan and collagen in tendon, (iii) knockdown studies, (iv) human patients with genetic disorder, and (v) gene variants responsible for susceptibility to tendon/ligament injury. The genes presented here are in mouse otherwise mentioned in human: (i) small leucine-rich proteoglycans: *Fmod*, *Lum*, *Dcn*, *Bgn*, and *ASPN* (human), (ii) matricellular: *Thbs2*, *Spp1, Sparc*, *Postn*, *TNC* (human), and *Tnxb*, (iii) other proteoglycans or glycoproteins: *Tnmd, Lect1, TNMD* (human),* Cd44, Prg4*, and *PRG4* (human), (iv) genes affecting proteoglycan responsible in tendon/ligament function: *Dse* and *Adamts5* and (v) other genes studied with proteoglycan or glycoprotein: *Col1a1* ^mov13^, *Col1a1* ^tm1Jae^, and human *COL27A1* (Table 1).

## 2. Small Leucine-Rich Proteoglycans (SLRPs) 

Small leucine-rich proteoglycans belong to the LRR superfamily of proteins. Members may contain up to 38 LRRs. The LRR domain is 20–29 amino acids long with asparagine and leucine residues in conserved positions [18]. The LRR is a structural motif used in the diverse molecular recognition processes. SLRP contains five-class (I–V) subfamilies (http://www.uniprot.org/uniprot): asporin, biglycan, decorin, and extracellular matrix protein 2 (class I); fibromodulin, keratocan, lumican, osteomodulin, and prolargin (class II); epiphycan, mimecan, and opticin (class III); chondroadherin, nyctalopin (class IV); podocan and podocan-like protein 1 (class V). SLRPs constitute a network of signal regulation being mostly extracellular and they are upstream of multiple signaling cascades. They affect intracellular phosphorylation, a major conduit of information for cellular responses, and modulate distinct pathways, including those driven by BMP/TGF*β* superfamily members, receptor tyrosine kinases such as ErbB family members, and IGFI receptor, and Toll-like receptors [18].

### 2.1. Fibromodulin (Fmod) and Lumican (Lum)

Fibromodulin and lumican are expressed in collagenous connective tissues and play role in establishing tissue integrity. The core protein of both has one LRRNT and 11 LRRs that are sites of protein-protein interactions. The keratan sulfate proteoglycan Lum is a major component of the corneal stroma. It is also widely expressed in the interstitial connective tissue matrices of the skin, tendon, and intestinal submucosa [19–21]. Fibromodulin is expressed in a number of tissues including higher levels in knee epiphysis, calvarial and diaphyseal bone, nasal and costal cartilage, eye, and the bladder [19, 20, 22]. Fibromodulin has been reported to express in tendon [23]. Between the Fmod and Lum, Fmod has higher affinity for the collagen due to its two-collagen binding sites, LRR-11 (higher affinity site) and LRR-7 (lower affinity site) [24]. Lumican has one collagen-binding site homologous to Fmod LRR-7 [25]. The two SLRPs compete for collagen binding via their LRR-7 site, but Fmod has a higher affinity for collagen due to the presence of an additional higher-affinity LRR-11. This relates to the physiological relevance during tendon development, when Lum is expressed early and Fmod later during the fibril assembly [26, Table I]. In addition, the abnormal collagen fibril morphology occurs early in Lum-deficient mice and late in Fmod-deficient mice [26]. Single or double mutants of these SLRPs exhibit varied range of abnormalities in collagen fibrillogenesis in Achilles, patellar, and FDL tendons indicating their requirement for tendon collagen fibrillogenesis [26–28]. Abnormalities in patellar tendon lead to OA disease in double mutants [28, Table I]. An increased content of non-cross-linked Col *α*2(I) was found in tendon from *Fmod* ^−/−^ mice. Fibromodulin may specifically target the cross-linking between *α*1(I) and *α*2(I) chains; however, in its absence, there is higher amount of cross-linked *α*1(I) chains. This change in cross-linking pattern may be due to the presence of other SLRPs, for example, higher Lum in *Fmod* ^−/−^ tendon [27, Table I]. The formation of mechanically strong fibrils may require Fmod-regulated crosslinking. The absence of Fmod would allow a higher activity of Lum that instead cross-links collagens into mechanically weaker fibrils [29].

### 2.2. Biglycan (Bgn) and Decorin (Dcn) and Association with Fmod, Lum, and Collagen

Biglycan and Dcn belong to the SLRPs class I subfamily. Both contain 12 LRRs. Biglycan has two attached GAG chains and Dcn has one. The GAG can be either chondroitin sulfate or dermatan sulfate depending on the tissue origin. Biglycan is found in several connective tissues, predominantly in articular cartilages. It is a homodimer and forms a ternary complex with microfibrillar-associated protein 2 and elastin and may be involved in collagen fiber assembly. Biglycan binds to Col I in the gap zone of the fibrils, and Dcn competes for that interaction [30]. One molecule of Dcn core protein interacts with four to six collagen molecules. Biglycan and Dcn bind to the same site in N-terminal region of collagen VI complex and compete for the same binding site [31]. Biglycan can connect collagen VI to collagen II [32]. 

#### 2.2.1. Biglycan (Bgn) and Decorin (Dcn)

**Figure 3:**
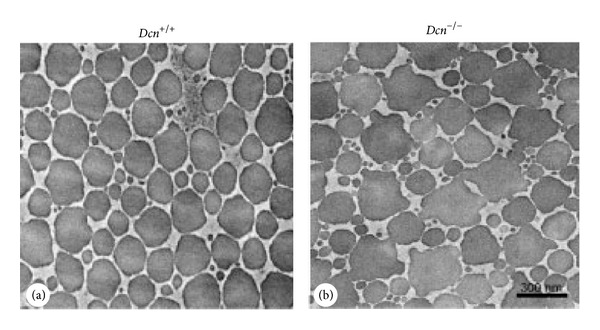
Decorin deficiency results in aberrant tendon collagen fibril structure. TEM of tail tendon of 7.5-month old mice showing transverse section of collagen fibrils. A larger fraction of fibrils are noncylindrical and structurally aberrant in homozygous mutant (*Dcn* ^−/−^, (b)) as compared to WT (*Dcn* ^+/+^, (a)) mouse (Reprinted from Zhang et al., 2006 [33], copyright © 2005 Wiley-Liss, Inc. with permission).

The effects of the two GAG chains present on Bgn are reasonably different from the single GAG of Dcn. The switch in expression levels of these SLRPs during tendon development is demonstrated by the fact that Dcn protein increases gradually with development from P4 to P30, whereas Bgn core protein decreases from P4 to P30 in flexor tendon. The stage in normal tendon development (P30), where Dcn peaked and Bgn decreased to its lowest level, indicates that Dcn persists until thick fibrils are formed [33, Table I]. The two GAG chains are proper organizers in the formation of early fibrils, perhaps by controlling the multitudes of small fibrils that would otherwise assemble in an uncontrolled manner. Biglycan, and not Dcn, is upregulated by 100% in compressed tendons where mechanical stress induces collagen fibrillogenesis [34]. Decorin-deficient mice showed abnormal collagen fibrils in FDL, and in tail tendon (Figure 3), the effect was more severe indicating the differential role of this proteoglycan in different tendons [33, Table I]. In the initial studies, when Danielson and coworkers knocked out the mouse for Dcn gene, they did notice similar severe phenotype in tail tendon collagen fibrils in Dcn-deficient mice [35, Table I] as also shown by Corsi and coworkers [36, Table I]. Reduced mechanical properties were recorded in FDL tendon in mature Dcn-deficient mice [33, Table I].

#### 2.2.2. Decorin (Dcn) and Periodontal Ligament (PDL)

Häkkinen and coworkers reported that Dcn plays role in maintaining structure and cellularity in PDL. In Dcn homozygote mutant mice, PDL collagen fibers are wider, random, and with varied small-sized fibrils. The mutation caused hypercellularity in PDL [37, Table I]. Ectopic overexpression of Dcn, in periodontal fibroblasts, suppressed the cell growth *in vitro*, indicating that Dcn regulates cell proliferation negatively in PDL [37, Table I]. Dourte and coworkers discovered that the viscoelastic and tensile dynamic modulus increased in the heterozygote (*Dcn* ^+/−^) tendons in which a decrease in collagen content was also discovered as compared to WT tendons [38, Table I]. In another interesting study, Ilkhani-Pour and colleagues showed that injured Dcn-deficient Achilles tendon heals better. Injured *Dcn* ^−/−^ tendons showed decreased tendon crosssection area, increased linear modulus, decreased tan (*δ*), and increased dynamic modulus |E*| compared to WT Achilles tendon. Authors suggested that the deletion of Dcn during tendon healing might have reduced scarring and improved collagen fibrillogenesis [39, Table I]. 

#### 2.2.3. Decorin (Dcn) Structure and Function Affected by Dermatan Sulfate Epimerase (Dse)

It is worth mentioning an enzyme, dermatan sulfate epimerase (Dse), that impacts the property of SLRP containing chondroitin sulfate/dermatan sulfate (CS/DS) by converting glucuronic acid unit to iduronic acid. Chondroitin sulfate is an unbranched polymer chain composed of alternating glucuronic acid and *N*-acetylgalactosamine units. In dermatan sulfate, D-glucuronic acid is converted to its epimer L-iduronic acid. The extent of this modification varies from a few percent of the glucuronic acid being epimerized to a predominant presence of iduronic acid and depends on the variable epimerase activity in tissues and on the core protein attached to the chain in CS/DS proteoglycans [2, 40]. The name CS/DS denotes the hybrid nature of the chain. The altered CS/DS chains carried by Dcn affects tendon fibrillogenesis. Collagen fibrillogenesis in tail tendon in *Dse* ^−/−^ mice was adversely affected with the presence of collagen fibrils with increased diameter [40, Table I]. A similar pattern in phenotype is observed in mice deficient in Dcn that showed fibrils with increased diameter in tail tendon [33, 35, 36].

#### 2.2.4. Lumican  (Lum), Fibromodulin (Fmod), Dbl-KO (Lum and Fmod), and Decorin (Dcn)

Teeth in SLRPs-deficient mice (*Lum* ^−/−^, *Fmod* ^−/−^, *Fmod* ^−/−^/*Lum* ^−/−^, and *Dcn* ^−/−^) erupted normally but histology and electron microscopy revealed abnormalities in PDL collagen fiber bundles. Lumican-deficient mice showed collagen fibers with smaller diameter and with varied interfibers spaces, fibromodulin-deficient fibers showed thicker bundles with poorly defined outlines [41, Table I]. On the other hand, in the mice deficient both in Fmod and Lum (*Fmod* ^−/−^/*Lum* ^−/−^), PDL contained some of the attributes from both the single-KO mice. Interestingly, Dcn-deficient mice alone demonstrated that the PDL fiber bundles were more disrupted with more width as compared to all the other three mutants (*Lum* ^−/−^, *Fmod* ^−/−^, and *Fmod* ^−/−^/*Lum* ^−/−^) [41, Table I].

#### 2.2.5. Decorin (Dcn) and Collagen I (Col I)

Investigations from Soslowsky laboratory showed the role of Dcn and collagen content on the tendon properties. In the first study, they reported how quasilinear viscoelastic properties of tendon are affected by their content [42, Table I]. The authors performed uniaxial tensile stress-relaxation experiment on tail tendon fascicles from mice at different developmental age and genotype groups, that is, 8-weeks *Dcn* ^−/−^, 8-weeks *Col1a1* ^mov13/+^ [43–45], 8-weeks control, and 3-weeks control mice. The viscoelastic properties demonstrated a larger and faster stress relaxation for Dcn-deficient mice, a smaller and slower stress relaxation for 3-weeks old mice with less collagen and more proteoglycan. The elastic parameter in 8-weeks control group was greater than the mice with reduced collagen (*Col1a1* ^mov13/+^) and with 3-weeks control [42, Table I]. Another study, from the same group, proved that the viscoelasticity of tendon fascicle is affected by Dcn content but not by collagen alteration [46, Table I]. In this study, Robinson and co-workers studied the mechanical properties of tail tendon fascicles in mice of different genotypes and age groups: 8-weeks *Dcn* ^−/−^, 8 wks *Col1a1* ^mov13/+^ (mice with 50% less Col I), 8-weeks Col1a1^tm1Jae^/Col1a1^tm1Jae^ (mice with accumulated Col I in soft tissues, [47, Table I]), 8-weeks control (normal mature mice), and 3-weeks control (immature mice with increased proteoglycan with GAGs in their tendon). Altered collagen in tail tendon fascicle in mice *Col1a1* ^mov13/+^ or *Col1a1* ^tm1Jae^/Col1a1^tm1Jae^ led to reduced failure load and stiffness with no changes in failure stress, modulus, or strain rate sensitivity. Decorin-deficient fascicles had similar elastic properties as normal control fascicles but with reduced strain rate sensitivity. Fascicles from immature mice had inferior elastic properties but higher strain rate sensitivity [47]. Using the similar set of mice, tendon fascicle structure/function relationship was established using multiple regression models and relative contributions of seven different structural and compositional variables in predicting tissue mechanical properties [48, Table I]. GAG content was observed to be the strongest predictor of mechanical properties and was also well correlated with collagen content and mean collagen fibril diameter. Collagen fibril area fraction was a significant predictor only of material properties. This concluded that in a large multivariate model, GAG content is the largest predictor of mechanical properties [48, Table I].

#### 2.2.6. Biglycan (Bgn) and Fibromodulin (Fmod)

By using Bgn-deficient mice [49, Table I], Corsi and co-workers were able to show altered collagen fibrillogenesis in tail tendon [36, Table I]. Tendon from 2 month male hemizygous *Bgn* ^−/0^ mice showed abnormal shaped fibrils, and with large diameters Bgn deficiency adversely affects the mechanical property of the healing bone insertion site of the patellar tendon fibers 4-weeks after surgery. Collagen fibril diameter distribution was disturbed in mutant mice [50, Table I]. Biglycan is expressed in PDL, alveolar bone (AB), at the AB-PDL attachment site, and at the cementum-PDL attachment site in mice. Histomorphometric analysis of X-ray *μ*CT images of Bgn-deficient cementum-PDL-AB complex exhibited abnormalities with higher PDL space, compromising the integrity of periodontal tissue [51, Table I]. Double homozygote mutants for Bgn and Fmod genes showed severe defects in mouse joint tendons that led the mouse to develop OA. Quadriceps tendon showed altered collagen fibrils in all the mutants (*Bgn*-KO, *Fmod*-KO, and Dbl-KO) but the severity of alteration was more in Dbl-KO. Reduced stiffness was noticed in patellar tendon at an early age [52, Table I]. In fact, Dbl-KOs developed premature OA and were at the predisposition to OA. The mutants represent a model for spontaneous OA, early-onset, and rapid progression of OA [52, Table I]. Working on the same Dbl-KO mice, there appeared an elegant study from Young's laboratory. The authors used patellar tendon from Dbl-KO (*Bgn* ^−/0^/*Fmod* ^−/−^) and demonstrated the existence of an ECM niche for tendon stem/progenitor cells (TSPCs). Patellar tendon in Dbl-KO mice was thinner and hypercellular, and exhibited disorganized collagen fibers and gaps [53, Table I]. The TSPCs from Dbl-KO mice presented higher number of colonies in cell culture when compared to TSPCs from WT mice indicating that the cells loose their “stemness” when isolated from mutant tendons. The authors hypothesized that an ECM-rich niche, organized by Bgn and Fmod, controls the self-renewal and differentiation of TSPCs in tendon [53, Table I]. In another study on these mutant mice, Kilts and colleagues showed that the mice deficient in Bgn, Fmod, or both developed ectopic ossification in tendon with aging in male and female mice. At 3-month old, all the mutants displayed torn cruciate ligaments and ectopic ossification in their quadriceps tendon, menisci, cruciate ligament, and patellar ligament; the phenotype was least severe in Fmod-deficient, intermediate in Bgn-deficient, and the most severe in Dbl-KO mice [54, Table I]. 

#### 2.2.7. Biglycan (Bgn) and Decorin (Dcn)

By using tendons from different loading regions of mutant mice deficient in Bgn and Dcn, Robinson and co-workers were able to demonstrate that tendons are tailored according to their location [55, Table I]. Mechanical properties of tail tendon fascicle did not show any change due to deficiency of either proteoglycan, whereas the loss of Dcn affected patellar tendon causing an increase in the modulus and stress relaxation but had little effect on FDL tendon. Dunkman and co-workers showed that aged patellar tendon has decreased dynamic modulus and viscoelastic property, decreased cellularity and alteration in tenocyte shape, and reduced collagen fibers alignment as compared to mature tendon [56, Table I]. Interestingly, Dcn-deficient tendons exhibited decreased effects of aging compared to the biglycan deficient or WT due to reduced detrimental effects on collagen fibrils [56, Table I]. Connizzo and co-workers studied realignment of collagen fibers and mechanical properties of aging supraspinatus tendons at 90–570 days of age in proteoglycan-deficient mice. The Bgn- or Dcn-deficient tendon showed altered mechanical properties with age, predominantly at the insertion site. Changes in realignment throughout age were not found in the midsubstance of the Bgn-deficient tendons or at the insertion of Dcn-deficient tendons. The study showed that Dcn and Bgn contribute to tendon's response to load, in particular with realignment of collagen fibers [57, Table I]. Both Bgn and Dcn have a role in the cornea fibrillogenesis [58]. Biglycan is upregulated in Dcn-deficient cornea and is considered to replace the function of Dcn. In contrast, Dcn reactivity was comparable in Bgn-deficient mice.

### 2.3. Asporin (Aspn)/Periodontal Ligament-Associated Protein 1 (PLAP-1)

Asporin contains 11 LRRs and 1 LRRNT domain. It is a critical regulator of TGF*β* in articular cartilage and plays an essential role in cartilage homeostasis and OA pathogenesis. Asporin blocks chondrogenesis and inhibits TGF*β*1-induced expression of matrix genes. Knockdown of Aspn increases the expression of cartilage marker genes and TGF*β*1, which, in turn, stimulates Aspn expression in articular cartilage cells, suggesting that Aspn and TGF*β*1 form a regulatory feedback loop. Asporin inhibits TGF*β*/Smad signaling upstream of TGF*β* type I receptor activation *in vivo* by colocalizing with TGF*β*1 on the cell surface and blocking its interaction with the TGF*β* type II receptor [59]. Asporin interacts with type I collagen. Decorin can inhibit collagen binding (Q99MQ4 at http://www.uniprot.org/). The LRR 5 repeat of Aspn can inhibit BMP2-induced cytodifferentiation. The induction of mutation in LRR5 within Aspn rescued the inhibitory effect of Aspn on BMP2 [60]. Asporin can bind collagen at the same site as Dcn, but it drives the biomineralization of collagen in contrast to Dcn and Bgn [61]. Asporin binds collagen type I. This binding is inhibited by recombinant Aspn fragment LRR 10–12 and by full-length Dcn, but not by Bgn. The polyaspartate domain of Aspn binds calcium and regulates hydroxyapatite formation *in vitro*. In the presence of Aspn, the number of collagen nodules as well as osterix and Runx2 mRNA, increased. Moreover, Dcn or the collagen-binding Aspn fragment LRR 10–12 inhibited the proosteoblastic activity of full-length Aspn. Thus Aspn and Dcn compete for binding to collagen and the polyaspartate in Aspn directly regulates collagen mineralization [61].

Asporin is expressed at higher level in the heart and specifically and predominantly in the PDL. During tooth development, strong expression is seen in the dental follicle, which is the progenitor tissue that forms cementum, alveolar bone, and the PDL [62, 63]. At E15.5, Aspn RNA expression is prominent in the developing mouse skeleton, particularly in the perichondrium/periosteum of cartilage/bone, and is found in other specialized connective tissues such as tendon, sclera, the connective tissue sheath surrounding muscle, and dermis [64].

Aspn knockdown studies showed that it negatively regulates PDL differentiation and mineralization to ensure that the PDL is not ossified, maintains homeostasis of the tooth-supporting system, and also inhibits Bmp2-induced cytodifferentiation of PDL cells by preventing its binding to BmpR1B, resulting in inhibition of Bmp-dependent activation of SMAD proteins. [62, Table I]. Li and coworkers reported that micro-RNAs miR-21 and miR-101 regulate Aspn expression in PDL cells. By using dual luciferase reporter assay and RNA expression assays, the group showed that miR-21 and miR-101 target Aspn to regulate its expression during osteogenic differentiation of PDL cells [65, Table I].

## 3. Matricellular Proteins

Matricellular proteins are extracellular matrix proteins that modulate cell-matrix interactions and cell function and do not seem to have a direct structural role. The family includes thrombospondin-1, thrombospondin-2, osteopontin/Spp1, osteonectin/Sparc, periostin, tenascin C, and tenascin X. Expression of matricellular proteins is usually high during embryogenesis, but nearly absent during normal postnatal life. Interestingly, it reappears in response to injury [66]. 

### 3.1. Thrombospondin 2 (Thbs2/Tsp2)

Thrombospondins are secreted, multimeric multidomain glycoproteins that function at the cell surfaces and the ECM and belong to thrombospondin family. They act as regulators of cell interactions in vertebrates. Thrombospondins consist of two subfamilies, A and B [67]. The subfamily A, proteins of Tsp1 (Thps1) and Tsp2 (Thbs2), assemble as homotrimer (P35441 and Q03350 at http://www.uniprot.org/uniprot/ resp.). The subfamily B of thrombospondins, consisting of Tsp3, Tsp4, and cartilage oligomeric matrix protein (Comp, also designated Tsp5), assemble as pentamers [67]. 

Thrombospondin 2 is a ligand for CD36 via the TSP-I repeats and delivers an antiangiogenic effect [68, 69]. It is homotrimeric and disulfide-linked and interacts (via the TSP type I repeats) with heregulin, and the interaction blocks the antiangiogenic effect of Tsp2 with CD36 [69]. Thrombospondin 2 can bind to fibrinogen, fibronectin, laminin, and collagen V [69–72]. Heparan sulfate proteoglycan, low-density lipoprotein receptor related protein, and *α*V*β*3 integrin have also been shown to be receptors for Tsp2 [69, 73, 74]. Adult mice do not express Tsp2 in collagen fibers of skin and tendon. Also tendon fibroblasts are not immune-reactive to Tsp2. Embryonic tendons, which are more cellular and grow rapidly, display high levels of Tsp2 transcript [75, 76]. Tsp2 is required for proper collagen fibrillogenesis in skin and tendon [77, Table I] and its absence disrupts fibroblast cell-matrix interaction during postnatal development of Tendon [78, Table I].

### 3.2. Secreted Phosphoprotein 1 (Spp1)/Osteopontin (Opn)/Bone Sialoprotein 1

Secreted phosphoprotein 1 is expressed in many tissues and cell types and found in body fluids [79]. The secreted protein is heavily modified posttranslationally by O-glycosylation, sulfation, and serine/threonine phosphorylation, the processes are heterogeneous and vary according to the cell origin [80, 81]. Spp1 is extensively phosphorylated on serine residues [82]. The functional domains of Spp1 are well conserved among species the central integrin attachment motif GRGDS, thrombin cleavage site, cryptic integrin attachment motif “SVVYGLR”, and mineral binding polyaspartate region. Many of the phosphorylated and glycosylated sites are well conserved [83].

Spp1 binds tightly to hydroxyapatite and forms an integral part of the mineralized matrix. It plays a role in cell-matrix interaction [84]. It acts as a cytokine involved in enhancing production of INF-*γ* and IL-12 and reducing IL-10 and is essential in the pathway that leads to type I immunity [84]. This cytokine and mineral matrix protein plays an important role in a number of physiological and pathological events, including tissue repair, regulation of bone metabolism, inflammation, and immunity [85]. Opn/Spp1 was highly upregulated during the muscle regeneration process induced by injection of the snake venom, cardiotoxin. SPP1 is expressed in the cells around calcified tendinitis. Spp1 plays role in the process of calcification of rotator cuff tendons [86]. Spp1 mRNA is expressed in normal patellar tendon of WT mice. By IHC, WT tendon expresses Spp1 protein in fibroblasts the latter exists in the interstitial space in tendon matrix [85]. Genetic deletion of *Spp*1 gene showed normal development [87, Table I]. However, *Spp*1^−/−^(*Opn* ^−/−^) mice demonstrated that Spp1 plays a role in stress-induced tendon remodeling [85, Table I].

### 3.3. Secreted Protein Acidic and Rich in Cysteine (Sparc)/Osteonectin (ON)

Sparc regulates the cell growth through interactions with the ECM and cytokines. It binds to calcium, copper, and several types of collagen, albumin, thrombospondin, PDGF, and cell membranes. There are two calcium binding sites: an acidic domain that binds 5 to 8 Ca^2+^ with low affinity and an EF-hand loop that binds a Ca^2+^ ion with high affinity. Sparc protein, secreted in ECM, is present in and around basement membrane and has been shown in mineralized and nonmineralized tissues [88]. It belongs to SPARC family and contains 1 EF-hand domain, 1 follistatin-like domain, and 1 Kazal-like domain. Sparc is expressed at high levels in tissues undergoing morphogenesis, remodeling, and wound repair [89]. It is a collagen-binding protein that has a great impact on ECM structure and function. It is glycosylated posttranslationally and is secreted in most tissues. Sparc is considered as a matricellular protein that modulates interactions between the cell and ECM and influences the efficacy of certain growth factors [90]. The Sparc-deficient mice revealed a function of Sparc in the deposition and accumulation of fibrillar collagen in tissues [89, 91]. Sparc plays role in pericellular processing of procollagen and functions in collagen turnover at the cell surface [91].

Collagen in the PDL has highest turnover rate in the body [92]. Hence, proteins that influence collagen deposition and turnover, such as Sparc, are expected to influence maintenance of structure and function of PDL. Sparc plays a role in human PDL disease, as demonstrated by its increased expression in gingival crevicular fluid of patients with periodontal disease [93]. Initial phenotype of *Sparc* ^−/−^ mice showed that Sparc is essential for the maintenance of lens transparency [94, Table I]. The Sparc-deficient mice suggest that Sparc function is related to collagen binding and to the regulation of ECM assembly and turnover. Specifically, Sparc-deficient mice had less collagen content in PDL [95, Table I]. Likewise, in lipopolysaccharide-induced inflammatory periodontal disease, Sparc-deficient mice lost more collagen in the PDL than the WT [96, Table I]. Based on their investigations [95, 96], Trombetta-Esilva and Bradshaw suggested a very convincing model of cellular mechanisms in that Sparc binds to procollagen the moment it is secreted from the cell (or procollagen is secreted already bound by Sparc) and prevents interaction of procollagen with cellular receptors, such as discoidin domain-containing receptor 2 (DDR2) and integrin *α*_2_ *β*_1_ or others. The procollagen is then appropriately processed and incorporated into collagen fibrils. In the absence of Sparc, procollagen accumulates at the cell surface and is inefficiently incorporated into the collagenous ECM, resulting in less total collagen and fewer thick collagen fibers [97].

### 3.4. Periostin (Postn)

Periostin has enhanced expression in periosteum and PDL. Periostin mRNA was shown to be upregulated at the sites under tension in bone and periodontal tissue remodeling after mechanical stress during experimental tooth movement [98]. It has been shown to play a role during developmental and wound repair [99]. Periostin mRNA expression level was higher during tendon graft healing process (112, 113). Periostin is a 90 kDa TGF*β*-induced secreted protein. It is a disulfide-linked protein. It is a member of fasciclin I family, which includes TGF*β*-induced protein and drosophila fasciclin I. Periostin domains have the following characteristics: the EMI domain binds to type I collagen, fibronectin, and Notch1, and the Fas I domains bind to tenascin-C and BMP-1. The C-terminal domain gives rise to splice variants and contains proteolytic cleavage sites [100].

**Figure 4:**
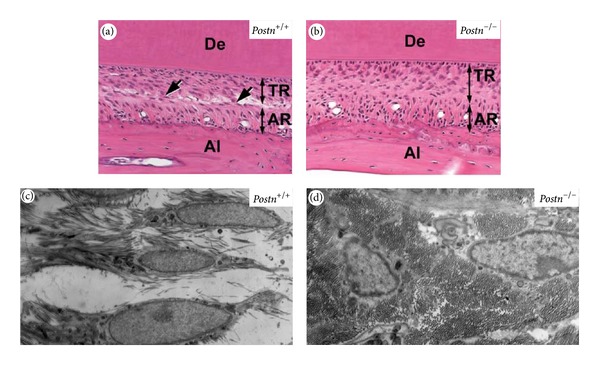
Remodeling of the PDL of incisors is defective in *Postn* ^−/−^ mice. H&E-stained PDL section from 12week *Postn* ^+/+^ (a) and *Postn* ^−/−^ (b) mice. Arrows indicate the shear zone, which is the boundary between the TR (tooth-related) and AR (alveolus-related) regions. The shear zone is clearly visible in 12 week old *Postn* ^+/+^ mouse (a) and absent in *Postn* ^−/−^ mouse (b). TEM of the incisor PDL shows evidence of digestion of collagen fibers in *Postn* ^+/+^ (c) but undigested and abundant collagen bundles in *Postn* ^−/−^ mice (d). De, dentin; Al, alveolar bone. (Reprinted from Kii et al., 2006 [102], with permission, Elsevier 2006, and thanks to Dr. A. Kudo for his generous approval.)

Periostin-deficient mice generated by Rios et al. [101, Table I] and by Kii et al. [102, Table I] both showed the fragility of the teeth due to defective PDL. In the embryonic teeth of the mouse mandible, Postn was localized to the interface between the inner enamel epithelium and preodontoblasts as well as in the mesenchymal tissues around the cervical loop [103]. At P7, the Postn protein was restricted to the fibrous bundles in PDL [104]. Postn is involved at sites of cell-to-matrix interaction, serving as an adhesive equipment for bearing mechanical forces including tooth eruption and transducing the occlusal force that activates latent TGF*β* to enhance Postn expression [105, Table I]. Consistently, *Postn* ^−/−^ mice showed defective eruption of their incisors [102, Table I]. The abnormal presence of nondigested collagen fibrils in the shear zone in the *Postn* ^−/−^ PDL [102, Table I, Figure 4] was explained by the low activity of matrix metalloproteinases, which are efficiently secreted following their induction by Postn [106]. Due to preferential expression of Postn in PDL and interaction with collagen fibrils, *Postn* ^−/−^ mice showed that Postn is required for the maintenance of the PDL integrity in response to mechanical stress [101, Table I], for the integrity and function of PDL during occlusal loading [104, Table I] and for the remodeling of incisors [102, Table I]. Other mutant *Postn* ^tm1Jmol^/*Postn* ^tm1Jmol^ mice, generated by Oka et al. [107], revealed that *Postn* ^−/−^ mice exhibit decreased cross-linking in tendon [108, Table I]. In another recent finding, WT mice tendon had higher failure load than *Postn* heterozygote mutants only but not than homozygote mutants during healing process [109, Table I].

### 3.5. Tenascin C (Tnc) and Tenascin X (Tnx)

Tenascins are a family of ECM proteins that evolved in early chordates [110]. There are four family members: tenascin X, R, W/N, and C. Tenascin X associates with type I collagen. The expression of tenascin C and tenascin W/N is developmentally regulated, and both are expressed during the disease state [110]. Tenascin C is a ligand for integrins *α*8/*β*1, *α*9/*β*1, *α*V/*β*3, and *α*V/*β*6 [111]. It is a homohexamer and disulfide-linked and is N-glycosylated [110]. It is expressed in nervous, skeletal, and vascular systems in embryonic stage and is involved in organ morphogenesis. In adult, tenascin C is expressed in dense connective tissues, smooth muscle, and stem cell niches of brain and bone marrow [110]. In disease, TNC is associated with asthma, fibrosis, wound healing, infection, tumor invasion, and metastasis [110]. Tenascin C is expressed during flexor tendon graft healing process during granulation phase [112, 113]. Tnc monomer in mouse comprises of 15 EGF-like domains, one fibrinogen C-terminal domain, and 14 fibronectin type-III domains (Q80YX1 at www.uniprot.org/uniprot/).

A large number of Achilles tendon injuries are associated with participation in sports [115]. Tenascin C is expressed in tendons [116]. In normal adult tendons, it is expressed predominately in regions transmitting high levels of mechanical force, such as the myotendinous and osteotendinous junctions [117, 118]. The protein is also expressed around the cells and collagen fibers of the Achilles tendon [119]. In addition, Järvinen and colleagues have shown that the expression of the TNC gene is regulated in a dose-dependent manner by mechanical loading in tendons [118, 119]. Isoforms of the protein, with distinct functions, are produced by alternative splicing of the primary transcript [103, 120, 121]. Healthy tendons express a small 200 kDa TNC isoform, while degenerate tendons express a functionally distinct larger 300 kDa isoform [103]. Ireland and coworkers have reported an increase in TNC expression in biopsy samples of chronic Achilles tendinopathies [122]. Human genetic studies show that chromosome 9q33 is involved in the predisposition risk of Achilles tendon injuries; for example, COL27AI rs946053, TNC rs13321, and TNC rs2104772 variants are significantly associated with risk of Achilles tendon injury in a South African and Australian subjects [123, Table I]. Earlier studies by Mokone and associates reported the association between a dinucleotide (GT) microsatellite marker within intron 17 of TNC and Achilles tendon injuries [124, Table I]. 

Tenascin X belongs to the tenascin family and contains 19 EGF-like domains, one fibrinogen C-terminal domain and 32 fibronectin type-III domains. Tenascin X mediates interactions between cells and the ECM. TNX is a large 450 kDa ECM protein expressed in a variety of tissues including skin, joints, and blood vessels. Deficiency of TNX causes a recessive form of EDS characterized by joint hypermobility, skin fragility, and hyperextensible skin. Skin of TNX deficient patients shows abnormal elastic fibers and reduced collagen deposition. TNX is homotrimer and interacts with type I, III, and V collagens and tropoelastin via its 29th fibronectin type-III domain [125]. It is highly expressed in fetal adrenal and testis, fetal smooth, and striated and cardiac muscle. Short isoform XB is only expressed in the adrenal gland. Expression levels are lower in adults than in children [126]. Tenascin X homozygote mutant mice showed skin hyperextensibility [127, Table I]. Further analysis of this mice revealed that tenascin X is required for force transmission of the myotendinous or myofascial pathways [128, Table I].

## 4. Glycoproteins, Adamts5, and Other Proteoglycans

### 4.1. Tenomodulin (Tnmd) and Chondromodulin-I (ChM-I)/Leukocyte Cell-Derived Chemotaxin 1

Tenomodulin is a member of a family of type II transmembrane glycoproteins. Tnmd transcripts have been found in hypovascular tissues such as tendons and ligaments but the biological activity of Tnmd has not yet been fully explored. Tnmd has been suggested to play a role in tendon development. The *Tnmd* gene is predominantly expressed in tendons, ligaments, and the eye [129, 130], but low levels of mRNA transcripts have been identified in some other tissues including cartilage [129]. Chondromodulin-I (encoded by *Lect1*) is a homologue of Tnmd and is highly expressed in cartilage and weakly expressed in some other tissues [129, 131, 132]. Each of the two proteins has distinct and overlapping expression pattern in tissues. Both the proteins contain two extracellular domains: BRICHOS and a C-terminal cysteine-rich domain [133]. The recombinant C-terminal cysteine-rich domain of ChM-I causes increased proliferation of primary chondrocytes [132], whereas endothelial proliferation was inhibited by the ectopic endothelial expression of the C-terminal cysteine-rich domain of either TNMD or ChM-I [134, 135]. 

Tenomodulin-deficient mice showed reduced cell numbers in adult tendons and a decrease in tenocyte proliferation at newborn stage indicating the role of Tnmd in tenocyte proliferation. In addition, the altered structure of adult collagen fibrils suggests an involvement of Tnmd in postnatal tendon maturation, though the angiogenesis was unchanged in tendons in Dbl-KO mice for Tnmd and ChM-I [136, 137]. The knockdown of tenomodulin in human flexor carpi radialis cells by RNAi approach reduced the cell proliferation and upregulated expression of myostatin and scleraxis indicating a potential negative feedback loop between TNMD and its regulators [138, Table I]. Scleraxis positively regulates the expression of TNMD, a differentiation marker of tenocytes [139].

### 4.2. Cd44 Antigen (Cd44)

CD44 is a single-pass type I membrane glycoprotein, also called hyaluronate receptor, and is a key mediator during normal wound healing, inflammation, and fibrotic healing process. The CD44 glycoproteins are members of hyaluronate receptor family of cell adhesion molecules. CD44 contains one extracellular lectin-like LINK domain that is responsible for hyaluronan binding. The major ligand is hyaluronate that is an abundant extracellular polysaccharide found in mammalian ECM, but CD44 has many varied functions depending on the extracellular structure of the protein, which can be produced in a myriad of isoforms. The wide range of functional proteins is produced from a single gene by both alternative splicing and post-translational modifications [150]. CD44 is N-glycosylated, O-glycosylated, and phosphorylated and contains chondroitin sulfate glycans that can be more or less sulfated. There are two allelic forms of this glycoprotein, PGP-1.1 and PGP-1.2. The expressed product is PGP-1.1 (Ly-24.1). One of the major roles of CD44 is to mediate the uptake and clearance of hyaluronate. 

Hyaluronic acid is abundant in tendons [151]. During adult tendon healing, CD44 and hyaluronic acid levels are elevated [152]. However, during scarless fetal healing, CD44 expression is downregulated [153] and hyaluronan levels surpass those of healing adult tendons. Higher levels of hyaluronic acid were shown to be beneficial in regenerative wound experiments [125]. Cd44-deficient mouse was generated by Tak Mak laboratory and the homozygous mutant mice showed abnormal hematopoiesis [140, Table I]. Interestingly, the study from Louis Soslowsky laboratory on these mutants further elaborated that injured patellar tendon heal better in Cd44-deficient mice than WT control mice [141, Table I] simulating the scarless fetal healing which occurs in the environment of low level of Cd44.

### 4.3. A Disintegrin and Metalloproteinase with Thrombospondin Motifs 5 (Adamts5) Cleaves Aggrecan

The ADAMTSs are a group of complex proteases found both in mammals and invertebrates. The complete human family has *≈*20 ADAMTS genes [154]. The ADAMTSs are extracellular multidomain enzymes with multiple functions: (i) collagen processing as procollagen N-proteinase, (ii) cleavage of the matrix proteoglycans aggrecan, versican, and brevican, (iii) inhibition of angiogenesis, and (iv) blood coagulation homoeostasis as the von Willebrand factor cleaving protease [154]. Adamts5 is comprised from N- to C-terminus: (i) a signal peptide, (ii) a prodomain, (iii) a metalloproteinase domain, (iv) a disintegrin domain, (v) a thrombospondin type motif (TSP Type-I), (vi) a cysteine-rich domain, (vii) a spacer region, and (viii) TSP type-I motif [149]. ADAMTS5 is a C- and O-glycosylated molecule. The spacer domain and the TSP type-1 domains are important for a tight interaction with ECM. The conserved cysteine present in the cysteine-switch motif binds to the catalytic zinc ion, inhibiting the enzyme. The dissociation of the cysteine from the zinc ion upon the activation-peptide release activates the enzyme (Q9R001 at www.uniprot.org/uniprot/). The precursor is cleaved by a furin endopeptidase. Adamts5 cleaves aggrecan, a cartilage proteoglycan, and is involved in its turnover. It plays an important role in the destruction of aggrecan in arthritic diseases and plays a role in proteolytic processing mostly during the peri-implantation period. Adamts5 cleaves aggrecan at the 392-Glu-*|*-Ala-393 site (Q9R001 at www.uniprot.org/uniprot/).

Aggrecan is most abundant in regions of tendon that experience mechanical compression and at enthesis [155]. Proteolytically degraded aggrecan has also been identified in tensional regions of normal adult bovine deep flexor tendons [156]. Disease ligaments from horses with degenerative suspensory ligament desmitis (DSLD) contain elevated levels of intact aggrecan and fragments generated by ADAMTS activity [157]. ADAMTS5 was abundant in the affected ligament but it was found to be complexed with hyaluronan around chondroid cellular clusters, and no active forms could be detected in these extracts. Genetically deleted mice for *Adamts5* cause aggrecan accumulation and affect the aggrecan turnover adversely. The accumulated aggrecan in tendon, in turn, affects the tendon functions adversely [142, 143].

### 4.4. Proteoglycan 4 (Prg4)/Lubricin/Superficial Zone Proteoglycan

Proteoglycan 4/lubricin plays a role in boundary lubrication within articulating joints, prevents protein deposition onto cartilage from synovial fluid, inhibits the adhesion of synovial cells to the cartilage surface, and prevents the articular chondrocytes apoptosis. Prg4 is a homodimer, disulphide-linked, and a secreted glycoprotein [158, 159]. It is highly expressed in cartilage, bone, and liver. It is expressed on the surface of chondrocytes and in synovial intimal cells [144, 160]. First detected, at the joint forming surface at E15.5 after cavitation, and at later stages of morphogenesis, strong expression is observed in superficial zone chondrocytes and in the newly forming synovium [144]. The molecule is N- and O-glycosylated and contains GAGs chondroitin sulfate and keratan sulfate [161, 162]. 

Different forms varying of Prg4 in molecular weight have been observed. Such forms are possibly due to different levels of glycosylation and protein cleavage. Prg4 contains two hemopexin-like domains and two somatomedin-B domains (Q9JM99 at www.uniprot.org/uniprot/). Prg4/lubricin is present in tendons, but its ability to improve tendon gliding was unknown until *in vitro *studies indicated that lubricin affects surface gliding and decreases gliding resistance [163]. Lubricin was found both on the flexor digitorum profundus tendon surface and at the interface of collagen fiber bundles within the tendon, where the cells are subjected to shear force in addition to tension and compression [164]. Six N-terminal splicing variants were identified from six distinct anatomical regions of flexor tendon. The variants with larger size were noted in regions subjected to significant shear and compressive forces [164]. The sheaths of the fascicles of the infraspinatus tendon near the bone-insertion site contain lubricin, indicating that this lubricating protein may be facilitating interfascicular movement. The fact, considering that the crimp pattern of fascicles changes with location in the tendon, provides support for the supposition that fascicles move relative to one another as the tendon is loaded, underscoring the importance of a lubricating protein in the layer separating the fascicles [165]. Mechanical loading has been shown to affect lubricin expression in flexor tendons, resulting in a 40% reduction of lubricin content in experimental non-weight-bearing flexor tendons [166]. 

Prg4/lubricin-deficient mice demonstrated abnormal calcification of tendon sheaths of tibialis anterior that surround the ankle joint. Absence of Prg4/lubricin within the tendon sheath results in decreased lubrication that led to tissue damage, matrix remodeling, and dystrophic calcification. These pathological changes led to camptodactyly similar CACP in human [144, Table I]. Lack of Prg4/lubricin did not affect Young's modulus in mice but rather it altered viscoelastic properties of tail tendon fascicles [146, Table I]. Prg4-deficient mice demonstrated abnormal calcification in joint tendons and sheaths leading to joint failure and that resulted in camptodactyly similar to human CACP patients [147, Table I]. Kohrs and colleagues demonstrated the reduction in lubrication in Prg4-KO mouse and the tail tendon exhibited increased intrafascicular resistance *ex vivo* [145, Table I]. In human patients with congenital camptodactyly and joint effusions, abnormalities in tendons were restricted to sheath [147, Table I]. Marcelino and colleagues showed that several mutations in human PRG4/lubricin are responsible for the cause for human CACP that alter the reading frame and result in premature truncation of the full-length polypeptide [148, Table I]. A deletion mutation was found in Prg4 gene in a family with CACP. Sequence analysis of Prg4 gene in the affected individuals revealed a 2-base-pair deletion predicting a frame shift mutation [149, Table I].

## 5. Perspectives

Understanding the mechanism of molecules in test tube is the first key step. Functional approach then can be better understood using the *in vivo* methods especially if genetic altered models are available and through knockdown approaches, availability of genetic variants predisposition to injury, and genes involved in human genetic diseases data. Proteoglycans and glycoproteins are part of tendon, ligament, and enthesis. Dry mass of tendon contains 1%–5% proteoglycans. This review has provided the functional importance of each molecule in tendon, ligament, or enthesis. Understanding these genes represents drug targets for disrupting pathological mechanisms that lead to tendinopathy, ligamentopathy, enthesopathy, enthesitis and tendon/ligament injury, that is, osteoarthritis.

Spondyloarthritis (SpA) refers to a group of HLA-B27-positive associated rheumatic diseases that share clinical and genetic features [167]. The diseases and conditions that constitute the SpA group are defined by signs, symptoms, and radiographic findings and are consisted of ankylosing spondylitis (AS), reactive arthritis, psoriatic arthritis, Crohn's disease, ulcerative colitis, and a subgroup of undifferentiated forms [168, 169]. The main clinical feature of SpA is inflammation of the axial spine. Articular, periarticular, and extra-articular manifestations also occur, depending on the type of SpA. Spondyloarthritis is a major health challenge given the propensity to affect young adults and the potential requirement for life-long treatment [170]. 

**Figure 5:**
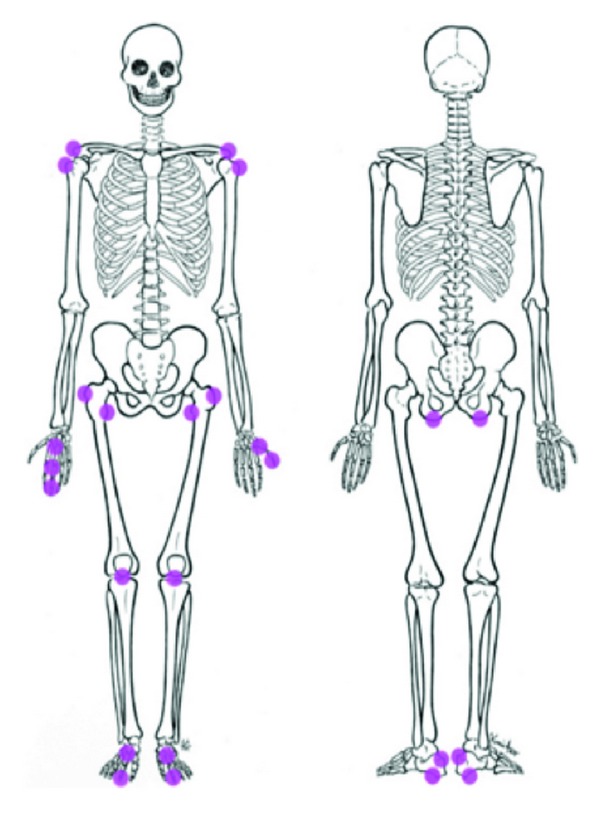
Most commonly affected appendicular entheseal sites in spondyloarthritis (Reprinted (with minor modification) from Eshed et al., 2007 [114], with permission, copyright © 2007, BMJ Publishing Group Ltd.)

A number of genes in this review have addressed the importance of proteoglycans and glycoproteins in joints in response to alteration in ligaments/tendon and hence via entheses. Abnormalities in entheses can lead to enthesitis. Inflammation at the entheses, the sites of attachment of tendon, ligament, fascia, or joint capsule to bone, is the distinguishing pathological feature of AS and the other SpA [171–173]. Ankylosing spondylitis is considered as a disease continuum with symptoms depending on age at onset and the important manifestation in the early stage of disease is not inflammatory back pain but peripheral arthritis and enthesitis [174]. Entheses are numerous and present everywhere, both in the axial and appendicular skeleton, explaining the wide clinical spectrum of enthesitis. Enthesitis can involve (a) synovial joints such as the sacroiliac joints, the zygaphophyseal joints, the hips, the shoulders, and the knees; (b) fibrocartilaginous joints such as the pubic symphysis, the intervertebral symphysis joint; (c) syndesmoses such as the interosseus sacroiliac ligament filling the irregular space posterosuperior to the sacroiliac joint; (d) extra-articular entheses [175]. The most commonly affected appendicular entheseal sites in SpA are shown in Figure 5 [114].

Excellent review has been published on a large number of gene variants involved in SpA and AS [170, 176]. Which genes are the cause and which are the effect still remains challenge in understanding the disease.

## Figures and Tables

**Table 1:** Tendon, enthuses, and ligament defects in response to genetic alterations: role of proteoglycans and glycoproteins.

Genetic mutation, altered variants, and knockdown approach	Tendon, ligament, and enthesis defects or predisposition to injury	Other tissues/organs affected, in brief and human disease	References
*Fmod* ^ tm1Aol^: fibromodulin; targeted mutation 1, Ake Oldberg Allele type: T (KO) Mut: insertion Syn: *Fmod* ^−^	Fibromodulin-deficient (*Fmod* ^−/−^) mice show abnormalities in tail and Achilles tendon. Histology revealed reduced number and disorganized fiber bundles with reduced number of cells in mutant tail tendon. Mutant mice also showed reduction in endotenon tissue. In WT tail tendon, the molar ratio of Lum : Fmod is 1 : 3. Fmod is expressed in collagen fibrils of tendon and Lum in peritendon area in tail and Achilles tendon. Lum protein level increased 4-fold in *Fmod* ^−/−^ tail tendon, whereas Dcn level did not change. Achilles tendon from fibromodulin-deficient mice showed collagen fibrils with irregular and rough outlines, altered fibril diameters, and disrupted fibril diameter frequency distribution across the tendon.	*Fmod* ^−/−^ mouse was generated. Mutant mice exhibit subtle phenotype, breed normally, and live normal life span.	[27]

*Lum* ^ tm1Chak^: lumican; targeted mutation 1, Shukti Chakravarti Allele type: T (KO) Mut: insertion (IGD) Syn: *Lum* ^−^	N/A	*Lum* ^−/−^ mouse was generated. Mutants have thicker collagen fibrils in skin and cornea and develop opacification. Mice show skin laxity resembling EDS, type I; OMIM: 130000.	[20]

*Lum* ^ tm1Chak^ *Fmod* ^tm1Aol^ Dbl-KO: *Lum* ^tm1Chak^ *Lum* ^tm1Chak^/ *Fmod* ^tm1Aol^ *Fmod* ^tm1Aol^ Or *Lum* ^−/−^/*Fmod* ^−/−^	Mice deficient in SLRPs (*Lum* ^−/−^, *Fmod* ^−/−^, and *Lum* ^−/−^/*Fmod* ^−/−^) show disruptions in collagen fibrillogenesis. Protein expression showed that Lum functions during early stages in fibrillogenesis, while Fmod functions throughout this period with a more prominent role in the regulation at later stages. The codistribution of Lum and Fmod was present throughout the tendon matrix at P10. Structurally, three distinct abnormalities were observed due to their deficiency: the premature heterogeneity of fibril diameter at P4 in *Lum* ^−/−^/*Fmod* ^−/−^ tendon in contrast to WT; abnormally large number of small diameter fibrils at later stages of development best seen at 3 months in *Fmod* ^−/−^ and *Fmod* ^−/−^/*Lum* ^−/−^ mice; all the three mutants had fibrils with irregular profiles and contours, indicative of abnormal defective lateral fusion and rearrangement. At 1–3 months, *Fmod* ^−/−^ and *Lum* ^−/−^/*Fmod* ^−/−^ mice had large number of abnormal “cauliflower-like” tendon fibrils with Dbl-KO fibrils showing the most severe phenotype. In contrast, *Lum* ^−/−^ tendons contained fibrils only slightly irregular in profile and contour.	The double homozygote mutant mice *Lum* ^−/−^/*Fmod* ^−/−^ were produced by Het × Het crossings and present mouse model for human EDS, type I; OMIM: 130000.	[26]
	Mice lacking both lumican and fibromodulin exhibit knee dysmorphogenesis, and extreme tendon weakness is the cause for joint laxity in mutant. Fmod deficiency alone leads to significant reduction in tendon stiffness but further loss in stiffness is demonstrated by the loss of Lum in a dose-dependent way. A disproportionate increase in small-diameter collagen fibrils in the *Fmod* ^−/−^ mice may be the cause of tendon weakness indicating that Fmod aids in fibril maturation. Dbl-KO mice display a 2-fold increase in knee joint deflection indicating increased joint laxity and show medial misaligned patella and secondary patellar groove in 65% cases. Dbl-KO mice show collagen fibrils with “cauliflower”-like contours in TEM cross-sections, indicating abnormal lateral growth in fibrils.	Double homozygote mutants *Lum* ^−/−^/*Fmod* ^−/−^ have smaller-sized body and develop age-dependent OA. The mice mimic human disease model: EDS, type I; OMIM: 130000.	[28]

*Dcn* ^ tm1Ioz^: decorin; targeted mutation 1, Renato V Iozzo Allele type: T (KO) Mut: insertion Syn: *Dcn* ^−^	Decorin-deficient mice exhibit abnormal collagen fibrillogenesis in tail tendon and a decrease in collagen-associated proteoglycans. Using TEM analysis, tendon fibrils showed irregular and ragged outlines in cross-sections in *Dcn* ^−/−^ mice compared to more uniform fibrils in *Dcn* ^+/+^ mice. The frequency distribution of fibril diameter across tendon was altered in *Dcn* ^−/−^ mice.	*Dcn* ^−/−^ mouse was generated which exhibits skin fragility and aberrant collagen fibrillogenesis and is a model for EDS, progeroid form; OMIM: 130070.	[35]
	Periodontal ligament is adversely affected by decorin deficiency in mouse. Using SEM, *Dcn* ^−/−^ molar PDL showed collagen fibers wider in diameter and more randomly arranged than WT. In TEM cross-sections of *Dcn* ^−/−^ molar PDL, the collagen fibers were irregularly shaped and were more randomly arranged. There was more heterogeneity in collagen fibers which constituted very large-diameter fibers with many very small-sized fibers in PDL in *Dcn* ^−/−^ mice. Molar PDL showed hypercellularity in mutants. Ectopic overexpression of exogenous Dcn, in PDL fibroblast cultures, suppressed cell growth suggesting decorin as a negative regulator of cell proliferation in PDL fibroblasts.	N/A	[37]
	Decorin deficiency alters structure and biomechanical properties of tendon during development. Bgn expression increased substantially in Dcn-deficient tendons suggesting a potential functional compensation. FDL tendon of *Dcn* ^−/−^ mice was analyzed at P1, 10, 60, and 90. Fibrils with irregular contour and lateral fusions were present at P10–P90. Besides FDL tendon, tail tendon exhibited severely altered fibril structure at the age of 7.5 months (Figure 3), also supporting that the defect in mutant mice is tendon specific. FDL tendon of mature mutant mice (P150) indicated a decrease in strength and stiffness.	N/A	[33]
	Patellar tendon retrieved from mature *Dcn* ^−/−^, *Dcn* ^+/−^, and *Dcn* ^+/+^ mice contains a different content of decorin and differs in its properties. Viscoelastic, tensile dynamic modulus increased in the *Dcn* ^+/−^ tendons as compared to *Dcn* ^+/+^. There was a reduction in total collagen in *Dcn* ^+/−^ tendon as compared to WT, though *Dcn* ^−/−^ and *Dcn* ^+/+^ tendons did not differ. Mean fibril diameter in *Dcn* ^−/−^ was smaller than WT and *Dcn* ^+/−^. The fibril diameter distribution histogram from *Dcn* ^−/−^ tendons demonstrated an increase in number of fibrils with smaller diameter. In TEM cross-sections, there was minor difference in the contour of fibrils in patellar tendon between different genotypes (unlike tail tendon). These results suggest that Dcn plays a role in tendon viscoelasticity that cannot be completely explained by its role in collagen fibrillogenesis. *Dcn* ^−/−^ tendons did not differ from WT in any tensile elastic parameter. In addition, no differences were seen in *Dcn* ^+/−^ compared to WT tendons in any of the elastic parameters. No differences were seen between genotypes for compressive properties. In the viscoelastic properties, Dcn genotype did not significantly affect phase shift that is a measure of the viscoelastic damping of the material. It is possible that the relationship between collagen, SLRPs, and water is more complex than evaluated here.	N/A	[38]
	Injured *Dcn* ^−/−^ Achilles tendons heal better than WT tendons. Four-month-old *Dcn* ^−/−^ mice underwent a bilateral, centralized, full-thickness, partial-width injury in Achilles, and animals were sacrificed at 3 weeks after injury. Uninjured *Dcn* ^−/−^ tendons had increased tendon cross-section area, decreased linear modulus, increased tan (*δ*), and decreased |E*| compared to WT, whereas injured *Dcn* ^−/−^ tendons had decreased tendon cross-section area, increased linear modulus, decreased tan (*δ*), and increased |E*| compared to WT. Deletion of Dcn during tendon healing may reduce scarring and improve collagen fibrillogenesis by allowing the formation of more mechanically stable collagen fibrils.	N/A	[39]

*Dse* ^ tm1Mmac^: dermatan sulfate epimerase; targeted mutation 1, Marco Maccarana Allele type: T (KO) Mut: insertion (IGD) Syn: *Dse* ^−^	The lack of Dse affects the epimerization of glucuronic acid to iduronic acid in glycosaminoglycans (GAGs) and affects the tendon properties. In tail tendon, in *Dse* ^−/−^ mice, the frequency distribution of collagen fibril diameter shifted towards larger-diameter fibrils, whereas in Achilles tendon, there were subtle changes compared to WT counterparts.	*Dse* ^−/−^ mouse was generated. Mice showed decreased litter size, birth length, and weight. Mice showed decreased skin tensile strength and thicker collagen fibrils.	[40]

*Dcn* ^ tm1Ioz^ *Fmod* ^tm1Aol^ *Lum* ^tm1Chak^	The SLRPs Lum, Fmod, and Dcn coordinately regulate the fibrillar organization of collagen in the PDL. These three SLRPs coexpressed with type I collagen in gingival and PDL. *Lum* ^−/−^ mice showed PDL phenotype different from WT and other SLRP mutants. In longitudinal sections, thin fiber bundles predominated. The fibers showed uneven outlines with numerous thin fibers projecting from the bundles. Individual collagen fibrils had irregular cross-sections and showed heterogeneity. Overall the fibrils diameter was smaller than other mutants and WT mice. Interfibrillar spaces varied. *Fmod* ^−/−^ PDL displayed collagen fiber bundles heterogeneous in thickness along the length. In contrast to *Lum* ^−/−^ mice, *Fmod* ^−/−^ PDL showed a relative increase in the number of thick fiber bundles. It was difficult to trace the fibril bundles from tooth to bone surface. Like *Lum* ^−/−^ but unlike WT mice, the spaces between the fibril bundles were not evenly distributed throughout the ligament. Individual collagen fibrils displayed enlarged cross-sectional areas, but with heterogeneity in the fibrils diameter, with small fibrils dispersed between larger ones. The inter-fibrils spaces were variable and enlarged. Fibril outline was uneven as compared to WT. *Fmod* ^−/−^/*Lum* ^−/−^ PDL contained some of the attributes of both single knockout mice. In *Dcn* ^−/−^ mice, the PDL collagen fiber bundles showed the typical 45° angle orientation seen in WT but the fiber bundles were heterogeneous in size with increased spaces between the bundles. The bundles were wider as compared to other three mutants and nonuniform in shape. Like in other mutants, the fibril bundles displayed very thin filamentous structures branching out from the main bundles. The fibrils were heterogeneous in size, with numerous small diameter fibrils among large diameter fibrils. The fibrils also displayed slightly uneven outlines as compared with WT. The inter-fibrils spacing was more variable as compared with WT.	N/A	[41]

*Col1a1* ^ mov13^: collagen, type I, *α*1; Moloney leukemia virus 13 Transgenic type: random, gene disruption Mut: viral insertion Inheritance: dominant Syn: *Col1a1* ^Mov13/+^	N/A	*Col1a1* ^ mov13/+^ mouse was generated [43], *Col1a1* ^mov13/mov13^ was reported to be lethal [44], and *Col1a1* ^mov13/+^ exhibited connective tissue defects and progressive hearing loss [45] and is a model for osteogenesis imperfecta type I; OMIM: 166200	[43–45]

*Dcn* ^ tm1Ioz^ *Col1a1* ^mov13/+^	Alteration in matrix proteins in tail tendon affects quasilinear viscoelastic properties. Uniaxial tensile stress-relaxation experiments were performed on tail tendon fascicles taken from mice at different developmental age and genotype groups: 8-week *Dcn* ^−/−^, 8-week *Col1a1* ^mov13/+^ (50% reduced type I collagen), 8-week control mice and 3-week control mice. The viscoelastic properties demonstrated a larger and faster stress relaxation for decorin-deficient mice and a smaller and slower stress relaxation for 3-week control mice (i.e., tendons with increased proteoglycans and associated GAGs). The elastic parameter in 8-week control group (tendon with more collagen but lesser proteoglycan than 3-week) was significantly greater than in the mice with reduced collagen (*Col1a1* ^mov13/+^) and 3-week control mice.	N/A	[42]

*Col1a1* ^ tm1Jae^: collagen, type 1, *α*1; targeted mutation 1, Rudolf Jaenisch Allele type: T (KI) Mut: insertion, nucleotide substitutions	N/A	*Col1a1* ^ tm1Jae^/*Col1a1* ^tm1Jae^ mouse was generated. Mutant mice are resistant to collagenase digestion and deposit increased collagen in skin.	[47]

*Dcn* ^ tm1Ioz^ *Col1a1* ^mov13^ *Col1a1* ^tm1Jae^	Viscoelasticity of tail tendon fascicle is affected by Dcn content but not by collagen alterations. Mechanical properties of tail tendon fascicles were assessed in different genotype and age group of mice: 8-week *Dcn* ^−/−^ (mouse lacking Dcn), 8-week *Col1a1* ^mov13/+^ (mouse with 50% reduction in Col I), 8-week Col1a1^tm1Jae^/Col1a1^tm1Jae^ (Col I accumulation in soft tissues), 8-week control (normal mature mouse), and 3-week control mice (immature mouse). Uniaxial tensile ramp to failure experiments were performed on tail tendon fascicles at two strain rates, 0.5%/s and 50%/s. Mutations in Col I (*Col1a1* ^mov13/+^ or *Col1a1* ^tm1Jae^/Col1a1^tm1Jae^) led to reduced failure load and stiffness with no changes in failure stress, modulus, or strain rate sensitivity. Dcn-deficient fascicles had similar elastic properties as normal control fascicles, but with reduced strain rate sensitivity. Fascicles from immature mice (3-week control with increased Dcn content compared to adult fascicles) had inferior elastic properties but higher strain rate sensitivity. It is evident that the tendon viscoelasticity is affected by proteoglycan Dcn content and not by collagen alterations.	N/A	[46]
	In a large multivariate model, glycosaminoglycan (GAG) content is the largest predictor of mechanical properties. Tendon fascicle structure-function relationship was established in transgenic models using multiple regression models. Relative contributions of seven different structural and compositional variables were used in predicting tissue mechanical properties through the use of multiple regression statistical models. Structural, biochemical, and mechanical analyses were performed on tail tendon fascicles from different groups of transgenic mice as shown in the last reference [46]. GAG content was observed to be the strongest predictor of mechanical properties. GAG content was also well correlated with collagen content and mean collagen fibril diameter. Collagen fibril area fraction was a significant predictor only of material properties.	N/A	[48]

*Bgn* ^ tm1Mfy^: biglycan; targeted mutation 1, Marian F. Young Allele type: T (KO) Mut: insertion Syn: *Bgn* ^−^	N/A	*Bgn*-KO mice were generated. Bgn-deficient mice show growth retardation and osteoporosis-like symptoms.	[49]
	Lack of biglycan adversely affects the mechanical property of the healing bone insertion site of the patellar tendon (PT) fibers. For that, the authors compared 12-week-old hemizygous *Bgn* ^−/0^ and WT male mice, and the midsubstance of the PTs of the mice was surgically removed (a 3 mm gap injury model), and the mice were allowed for a cage activity after surgery for 4 weeks. The tensile test was performed for the healing patellar tendon-tibia complex. Collagen fibril diameter distribution was disturbed in mutant mice. At the beginning of surgery, frequency distribution of collagen fibril diameter was towards left (smaller diameter fibrils) in *Bgn* ^−/0^ as compared to WT tendon. At 4 weeks after surgery, the collagen fibril diameter distribution was towards right (larger diameter fibrils) in *Bgn* ^−/0^ as compared to 4 weeks after surgery in WT controls.	N/A Note that biglycan is located on X chromosome. Male biglycan deficient is represented as hemizygote *Bgn* ^−/0^, whereas female homozygote is represented as *Bgn* ^−/−^	[50]
	Lack of biglycan compromises the integrity of periodontal tissue. IHC of SLRPs indicated that Bgn is expressed in PDL, alveolar bone (AB), at the AB-PDL, and cementum-PDL attachment sites in WT mice. Histomorphometric analysis of *μ*XCT images of periodontal tissues of 8-week-old mice was performed. Deeper AB resorption pits within interdental region of *Bgn*-KO specimens were compared to WT which showed the significant difference in PDL space of *Bgn*-KO (93 mm^3^) and WT (74 mm^3^) mice. Cementum-PDL-AB complex exhibiting higher PDL space in *Bgn*-KO mice does compromise the integrity of periodontal tissue.	*μ*XCT analysis showed higher volumes of enamel, root, and larger tooth size in *Bgn*-KO than WT mice.	[51]
	Abnormal collagen fibrils in tendons of Bgn/Fmod-deficient mice lead to gait impairment, ectopic ossification, and osteoarthritis. Collagen fibrils in tendons from Dbl-KO mice are structurally and mechanically altered resulting in unstable joints. The mice progressively develop gait impairment, tendon ectopic ossification (EO), and severe premature OA. Daily forced running of *Bgn* ^−/0^/*Fmod* ^−/−^ mice for a month increased EO and resulted in severe OA. Radiographs of 3-month-old mice revealed EO in the Achilles, patellar, and quadriceps tendons of the knee in all the mutants (*Bgn*-KO, *Fmod*-KO, and Dbl-KO). Quadriceps tendon showed irregular collagen fibrils in cross-sections in all the mutants at the age of 3 months. Stiffness reduced significantly in patellar tendon in Dbl-KO at the age of 1 month before tendon begins to ossify.	Bgn and Fmod double deficient mice exhibit premature OA and predisposition to OA. The Dbl-KO mouse represents a model for spontaneous OA, early onset and rapid progression of OA.	[52]
	Mice deficient in biglycan and fibromodulin (*Bgn* ^−/0^/*Fmod* ^−/−^) show patellar tendon defects. Gross morphology of patellar tendon at the age of 4 months showed abnormal translucent color in *Bgn* ^−/0^/*Fmod* ^−/−^ mice, whereas tendon was white in color in WT mice. H&E of longitudinal sections of P6 tendon from Dbl-KO was more thinner, more cellular, and with more gaps among collagen fibers. Under polarized light, these exhibited disorganized collagen fibrils. *µ*CT of knees of 2-month and 5-month *Bgn* ^−/0^/*Fmod* ^−/−^ mice showed ectopic ossicles formed in patellar tendons.	N/A	[53]
	Mice deficient in biglycan and fibromodulin develop ectopic ossification (EO) in tendon with aging. At the age of 3 months, *Bgn*-KO, *Fmod*-KO, and Dbl-KO displayed torn cruciate ligaments and EO in their quadriceps tendon, menisci, cruciate, and patellar ligaments, and the phenotype was least severe in *Fmod*-KO, intermediate in *Bgn*-KO, and the most severe in Dbl-KO. The EO progressed with age from 3 months to 9 months. In Dbl-KO mice subjected to moderate treadmill exercise, the EO was decreased compared to “unexercised” mice. Male *Bgn* ^−/0^/*Fmod* ^−/−^ mice had more EO compared to female *Bgn* ^−/−^/*Fmod* ^−/−^ mice but both had decreased EO after the forced moderate treadmill exercise regime. Dbl-KO and *Bgn*-KO mice exhibited reduced ability to maintain grip on a rotating cylinder in rotarod performance test. At early stage of mineralization of tenocytes (in *Bgn*-KO or Dbl-KO) in mutant mice, the tenocytes lost their elongated fibroblast-like phenotype and acquired a rounded chondrocyte-like phenotype. With time, the intercellular spaces became completely mineralized, but the mineralization remained confined to the fibrocartilage area within the tendon in mutants. The mineralization process was also observed in the cruciate ligament of 3-month *Bgn*-KO and Dbl-KO.	N/A	[54]

*Bgn* ^ tm1Mfy^ *Dcn* ^tm1Ioz^ Dbl-KO: *Bgn* ^−/0^/*Dcn* ^−/−^(male)	Collagen fibrillogenesis is altered in tail tendon of *Bgn* ^−/0^ and *Dcn* ^−/−^ mice as demonstrated by collagen fibril morphology with frequent occurrence of irregular cross-sectional profile with ragged or notched contour. The range of collagen fibril diameter increased in mutant mice as compared to WT mice.	*Bgn* ^−/0^, *Dcn* ^−/−^, and *Bgn* ^−/0^/*Dcn* ^−/−^ mice showed varied defects in dermal and bone collagen fibrils. The *Bgn* ^−/0^/*Dcn* ^−/−^ mice mimic rare progeroid variant of EDS; OMIM: 130070.	[36]

*Bgn* ^ tm1Mfy^ *Dcn* ^tm1Ioz^	Tendons are tailored according to their specific location and function. Mechanical properties of tail tendon fascicles, patellar tendon (PT), and FDL tendon, each differing in their *in vivo* loading environment from one another, were characterized in 8–10-week-old Bgn- and Dcn-deficient mice. No change in mechanical properties was observed for tail tendon fascicles due to either proteoglycan's deficiency. The loss of Dcn caused an increase in modulus and stress relaxation but had little effect on FDL. Conversely, the loss of Bgn did not affect PT but caused a reduction in maximum stress and modulus of the FDL.	N/A	[55]
	Decorin deficiency protects aged tendons. Multiple properties of patellar tendon (PT) from mature (age, 150 days) and aged mice (age, 570 days) were studied in *Dcn*-KO and *Bgn*-KO mice. Aged WT PT exhibited inferior properties as compared to mature WT and showed deteriorating viscoelastic properties, reduced dynamic modulus, decreased cellularity, alterations in tenocyte shape, and reduced collagen fibers alignment. Fibril diameter distribution indicated an altered distribution in aged tendons with an increase of large-diameter fibrils. Dcn-deficient tendons exhibited decreased effects of aging compared to the other genotypes (*Bgn*-KO or WT). The amelioration of the functional deficits seen in the absence of Dcn in aged tendons was associated with altered tendon fibril structure which was comparable to mature WT tendon.	N/A	[56]
	Decorin and biglycan contribute to tendon's response to load, in particular with realignment of collagen fibers. Isolated supraspinatus tendon (SSTs) from Bgn- and Dcn-deficient mice, at three different age groups (90 days, 300 days, and 570 days), were tested in tension. Changes in realignment in WT tendons showed a decreased response to load with aging. Tendons at 300 days and 570 days do not realign their collagen fibers until the linear region, a later response than 90-day tendons. This result in WT tendon midsubstance is indicative of a breakdown of the structural organization of tendon over time. The proteoglycan-deficient tendons showed altered mechanical properties with age, predominantly at the insertion site. However, changes in realignment throughout age were not found in the midsubstance of the Bgn-deficient tendons or at the insertion of Dcn-deficient tendons. Lack of these proteoglycans may shield the tendon from deteriorating effects. Changes in mechanical properties did not occur in concert with changes in collagen fiber realignment, suggesting that typical mechanical property measurements alone are not sufficient to describe how structural alterations affect tendon's response to load.	N/A	[57]

*ASPN*: asporin Strategy: knockdown Syn: PLAP-1 (PDL-associated protein 1)	Knockdown of *ASPN* transcript levels by RNAi enhanced Bmp-2-induced differentiation of PDL cells. Overexpression of asporin in mouse PDL-derived clone cells inhibited both naturally and BMP-2-induced mineralization of the PDL cells, whereas knockdown of asporin transcript levels by RNAi enhanced Bmp-2-induced differentiation of PDL cells.	N/A	[62]

*ASPN* microRNA (miR) approach	MicroRNAs, miR-21, and miR-101 regulate asporin/PLAP-1 expression in PDL cells. Bioinformatic analysis predicted miRNAs that potentially regulate the gene expression of asporin. Dual luciferase reporter assay and qRT-PCR showed the effects of miR-21 and miR-101 on asporin gene expression. The results indicated that miR-21 and miR-101 target asporin to regulate its expression during osteogenic differentiation of PDL cells.	N/A	[65]

*Thbs2* ^ tm1Bst^: thrombospondin 2; targeted mutation 1, Paul Bornstein Allele type: T (KO) Mut: insertion (IGD) Syn: *Tsp2* ^−^	Lack of thrombospondin 2 results in connective tissue abnormalities associated with disordered collagen fibrillogenesis. Tail tendon exhibited abnormalities in *Thbs2* ^−/−^ mouse. The mutants show poor control of tail movement, when held by the tip of the tail, *Thbs2* ^−/−^ mice had difficulty in pulling their body up to the base of tail indicating that the mutant mice had abnormalities in tail tendon and in intervertebral ligaments. Tail tendon exhibited abnormalities in collagen fibrils presenting larger diameters and uneven contours in mutant mice (large fibril diameter range in mutant versus WT was in the order of 400 nm versus 250 nm, resp.).	*Thbs2* ^−/−^ mice were generated and showed reduced skin strength, abnormal collagen fibrillogenesis, bone and bleeding defects, and increased blood vessels.	[77]
	Thrombospondin 2 modulates cell-matrix interaction during postnatal development of tendon. Developing hind limb flexor muscle tendon showed abnormalities in fibroblast-collagen fibril interaction in *Thbs2* ^−/−^ mice. During tendon development, P4 and P8 tendons show fibroblastic extracytoplasmic channels where fibrils assemble first and then coalesce into small fibers. The “compartmentalization of fibrils assembly” as well as “of coalescence” was disrupted in developing tendon in *Thbs2* ^−/−^ mice.	*Thbs2* ^−/−^ mice showed abnormal skin collagen fibrils. The mutants also exhibited increased vascular density in skin.	[78]

*Spp1* ^ tm1Rit^: secreted phosphoprotein 1; targeted mutation 1, Susan R. Rittling Allele type: T (KO) Mut: Insertion Syn: *Spp1* ^−^, *Opn* ^−^, *Osteopontin* ^−^	N/A	*Spp1* ^−/−^ mouse was generated. The mutants showed altered osteoclastogenesis *in vitro*.	[87]
	Spp1 plays role in tendon remodeling after denervation-induced mechanical stress deprivation. Six-week-old males demonstrated mechanical stress deprivation in quadriceps femoris muscle after femoral nerve denervation. Patellar tendon underwent dynamic remodeling as evidenced by collagen fibril degradation. Transient upregulation of SPP1 expression was observed in early phase followed by induction of MMP13 during patellar tendon remodeling in WT animals, the later involved degradation of collagen. MMP-13 gene expression increased 20.7-fold at day 14 after stress deprivation in WT mice and 4.1-fold in mutants. Thus Spp1 plays a crucial role in conveying the effect of denervation-induced mechanical stress deprivation to the tendon fibroblasts to degrade ECM by regulating MMP-13 expression.	N/A	[85]

*Sparc* ^ tm1Hwe^: secreted protein acidic and rich in cysteine; targeted mutation 1, Chin Chen Howe Allele type: T (KO) Mut: insertion Syn: *SP* ^−^, *Osteonectin* ^−^, *ON* ^−^, *Sparc* ^−^	N/A	*Sparc* ^−/−^ mice were generated. Mutants develop opacities in posterior cortex of eye as early as 1.5 month after birth indicating that Sparc is essential for maintenance of lens transparency.	[94]
	Sparc plays a role in controlling collagen content in PDL and is required for PDL homeostasis. Sparc expression, evaluated at 4 time points of aging, In the highest levels were at the age of 1 month and >18 months and reduced levels at 4 months and 6 months in mouse PDL. Absence of Sparc influenced cellular and fibrillar collagen content in PDL. The greatest differences in cell number and in collagen content between *Sparc* ^−/−^ and WT PDL coincided with ages at which levels of Sparc expression were the highest in WT at 1 and >18 months.	N/A	[95]
	Sparc protects collagen content in PDL and alveolar bone in experimental periodontal disease. Periodontal disease was induced in 4-month WT and *Sparc* ^−/−^ mice by LPS injections between first and second molars. In LPS-injected sites, PDL of Sparc-deficient mice showed more disorganization compared to WT. Substantial degradation was shown in the gingival tissues in *Sparc* ^−/−^ mice. Collagen loss, induced by LPS as determined by “total collagen volume fraction” and “thick collagen volume fraction,” was more extensive in *Sparc* ^−/−^ mice than WT. In PBS-injected sites, Sharpey's fibers were thicker in WT but were more in number in mutants.	*μ*CT analysis of Sparc-deficient maxillae injected with lipopolysaccharide (LPS) demonstrated reduced alveolar bone volume fraction compared to WT mice.	[96]

*Postn* ^ tm1Sjc^: periostin, osteoblast specific factor; targeted mutation 1, Simon J. Conway Allele type: T (R) Mut: insertion (IGD) Syn: *Peri* ^lacZ^, *Postn* ^−^	Periostin is required for the maintenance of PDL integrity in response to mechanical stress. *Postn* ^−/−^ mice showed the formation of dental alveolar defects and severe incisor enamel defects by 3 months. Placing the *Postn* ^−/−^ mice on a soft diet alleviated mechanical strain on PDL, which resulted in a partial rescue of both the enamel and periodontal disease-like phenotypes. It was concluded that a healthy PDL is required for normal amelogenesis and Postn is required for maintaining the integrity of the PDL in response to mechanical stress. Postn showed higher expression in PDL than in other tissues in 4-week WT mouse, and *Postn* ^−/−^ mice showed abnormal PDL morphology at that age. Fully erupted molars displayed widening of the *Postn* ^−/−^ PDL. By the age of 3 months, *Postn* ^−/−^ mice develop an early-onset periodontal disease-like phenotype resulting from abnormalities in PDL.	*Postn* ^−/−^ mouse was generated, 14% mutants die before weaning, and the rest were runted. Trabecular bone in adult is sparse. Female cyclicity is abnormal. The *Postn* ^−/−^ represents periodontitis, aggressive 2, OMIM: 608526.	[101]
	Periostin is essential for the integrity and function of the PDL during occlusal loading. PDL integrity is required for periodontium structure function. Before tooth eruption, PDL is normal in *Postn* ^−/−^ mice. After eruption, sustainment of occlusal load became evident, and the lack of PDL integrity became obvious in mutants leading to alveolar bone defects and malformed incisors. Severe periodontal defects were observed in mutants after tooth eruption. With time, periodontium deteriorates and widening of PDL persisted in mutants. The removal of masticatory forces in mutants, by using occlusal hypofunction model, rescued the periodontal defects.	Alveolar bone, cementum, and enamel are affected adversely in *Postn* ^−/−^ mouse. The mutant mouse represents human disease: periodontitis, aggressive 2, OMIM: 608526.	[105]

*Postn* ^ tm1Kudo^: periostin, osteoblast specific factor; targeted mutation 1, Akira Kudo Allele type: T (KO) Mut: insertion Syn: *periostin* ^−^, *Postn* ^−^	Periostin is an ECM protein required for the eruption of incisors in mice. Remodeling of PDL of incisors is defective in *Postn* ^−/−^ mice. Continuous eruption of the incisors, by constant formation of dentin and enamel in mouse, is accompanied by the formation of a shear zone within PDL at which the PDL is remodeled continuously. The remodeling shear zone at which periostin is expressed in WT is shown to be absent in 12-week *Postn* ^−/−^ mice (Figure 4).	*Postn* ^−/−^ mouse was generated. Mutants exhibit abnormal ameloblast, dentin, and enamel morphology, have short incisors assessed at 6 weeks, and show abnormal tooth eruption.	[102]

*Postn* ^ tm1Jmol^: periostin, osteoblast specific factor; targeted mutation 1, Jeffery D. Molkentin Allele type: T (KO) Mut: insertion (IGD) Syn: Pn^−^, periostin^−^, *Postn* ^−^	N/A	*Postn* ^−/−^ mouse was generated and revealed that periostin regulates cardiac hypertrophic response, interstitial fibrosis, ventricular remodeling, and myocardial infarction.	[107]
	Periostin regulates collagen cross-linking in tendon. Periostin-deficient mice show decreased cross-linking in tendon. IHC and immunogold TEM demonstrated that periostin colocalizes with collagen type I in tendon. Using differential scanning calorimetry on samples from 3-month-old mice, lower denaturation temperatures for *Postn* ^−/−^ tendon, indicated reduced collagen cross-linking as compared to WT tendon.	*Postn* ^−/−^ mice exhibited skin and atrioventricular connective tissue defects.	[108]

*Postn*: periostin Species: Mus musculus Origin: not available Syn: *pstn* ^−^	Complete deficiency of periostin does not appear to affect failure load in intact as well as in healing Achilles tendon, whereas partial deficiency does. Role of periostin was investigated on intact and healing Achilles tendon using mouse deleted in *Postn/Pstn* gene. Achilles tendon from 10-week-old WT, *Postn* ^−/−^, and *Postn* ^+/−^ was transected and repaired. After 14 days of healing, failure load values in WT tendon were significantly higher than *Postn* ^+/−^ but nonsignificantly different from *Postn* ^−/−^ tendon. Similar results were observed in intact tendon among the three genotypes. Authors concluded that there must be some compensatory mechanism in homozygote mutant tendon in which failure load values are more close to WT.	N/A	[109]

*TNC* (*Homo sapiens*): tenascin C Loc: Chr 9q33 *COL27A1* (*Homo sapiens*): collagen, type XXVII, *α*1 Loc: Chr 9q32	Genetic variants within *TNC* gene and *COL27A1* genes on human chr9q32-33 are involved in Achilles tendinopathy (AT). *COL27A1* gene has been mapped to chromosome 9q32-33, 708 kbp upstream of *TNC*. In a case control study, 339 healthy control participants and 179 participants clinically diagnosed with AT from South Africa and Australia, were genotyped for variants: rs4143245, rs1249744, rs753085, rs946053 (*COL27A1*) and rs13321, rs2104772, and rs1330363 (*TNC*). The rs2104772 and rs1330363 variants within *TNC* showed a significant allele association with AT. The GCA haplotype (rs946053-rs13321-rs2104772) occurred significantly more frequently in 10 AT participants compared to control. The haplotype (i) rs946053 (G > T) lays within a putative c-Myc transcription factor binding site which is eliminated in the presence of the T-allele; (ii) rs13321 (G > C) lays within a putative GATA transcription factor binding site, and putative splicing regulatory elements are found 7 and 15 bp down stream of this SNP.	N/A	[123]

*TNC* (*Homo sapiens*): tenascin C Loc: Chr 9q33 Mut: guanine-thymine dinucleotide (GT) repeat polymorphism in intron 17	The GT repeats polymorphism within *TNC* gene is associated with Achilles tendon injuries. In a case control study, 114 physically active white subjects with symptoms of Achilles tendon injury and 127 asymptomatic physically active white control subjects were genotyped for GT repeats within *TNC* gene. Allele containing 12 and 14 GT repeats was overrepresented in subjects with tendon injuries, while the allele containing 13 and 17 GT repeats was underrepresented. Subjects with variants of *TNC* gene for 12 and 14 GT repeats had 6-fold risk of developing Achilles tendon injuries.	N/A	[124]

*Tnxb* ^ tm1Jbrs^: tenascin XB; targeted mutation 1, James Bristow Allele type: T (R) Mut: insertion (IGD) Syn: Tnx-KO, *Tnxb* ^−^	N/A	*Tnxb* ^−/−^ mouse were generated, and the skin showed hyperextensibility, failure of fibroblast to deposit collagen I. The mutant mouse is modelled for human EDS, autosomal recessive; OMIM: 606408.	[127]
	Tnx deficiency alters properties of force transmission pathways of muscle, the later being the part of myotendinous or myofascial pathways, and directly affects muscle function in *Tnxb* ^−/−^ mice.	N/A	[128]

*Lect1* ^ tm1Ref^: leukocyte cell derived chemotaxin 1; targeted mutation 1, Reinhard Fassler Allele type: T (R) Mut: insertion (IGD) Syn: *Lect1* ^−^, ChM-I chondromodulin	N/A	Chondromodulin-I-deficient (*Lect1* ^−/−^) mice were generated and showed no abnormal phenotype.	[137]

*Tnmd* ^ tm1Ref^: tenomodulin; targeted mutation 1, Reinhard Fassler Allele type: T (KO) Mut: insertion Syn: *Tnmd* ^−^ *Lect1* ^tm1Ref^	Tenomodulin is a regulator of tenocyte proliferation and is involved in collagen fibril maturation. Tnmd-deficient Achilles tendon exhibits lower cell density at 1 month and 6 months of age. Patellaris and Achilles tendon show reduced cell densities in 2 weeks but not in newborn or at P7 *Tnmd* ^−/−^ mice. Reduction in cell proliferation rate, in mutant patellaris tendon, was demonstrated in newborn. This deficit in cell proliferation rate caused lower cell density at P14 and adult tendons. At 6 months mutant tendons had a greater heterogeneity of collagen fibrils with more thick fibrils and fibrils with uneven surfaces.	*Tnmd* ^−/−^ mouse was generated. Mice lacking both Tnmd and ChM-I (*Tnmd* ^−/−^/*Lect1* ^−/−^) had normal retinal vascularization and neovascularization.	[136]

*TNMD* (*Homo sapiens*) Knockdown of all the three isoforms of TNMD by RNAi assay	Tenomodulin regulates cell proliferation. Targeting TNMD by RNAi assay (with siRNA against human TNMD exon 5) in human flexor carpi radialis (FCR) cells knocked down all the three human TNMD isoforms. The knockdown of FCR cells showed reduced cell proliferation and upregulated expression of myostatin and scleraxis.	N/A	[138]

*Cd44* ^ tm1Mak^: Cd44 antigen; targeted mutation 1, Tak W. Mak Allele type: T (KO) Mut: insertion (IGD) Syn: *Cd44* ^−^	N/A	*Cd44* ^−/−^ mouse was generated and showed altered tissue distribution of myeloid progenitors.	[140]
	Lack of Cd44 antigen leads to improved healing in injured patellar tendon. The study was conducted in a patellar injury model of 12-week-old *Cd44* ^−/−^ mice. The material properties of the healing *Cd44* ^−/−^ tendon were superior to WT at 3 weeks and 6 weeks after injury. The matrix components and cytokines, beneficial to healing, increased in mutant tendon. Cross-sectional area of tendon was significantly reduced in mutant tendon at 3 weeks and 6 weeks after injury.	N/A	[141]

*Adamts5* ^Δexon2^: a disintegrin-like and metallopeptidase (reprolysin type) with thrombospondin type 1 motif, 5; Exon2 deleted Allele type: T (KO) Mut: insertion (IGD) Syn: *Adamts5* ^−^	N/A	*Adamts5* ^−/−^ mouse was generated. Mice were resistant to destabilization of the medial meniscus-induced OA-like lesions and to the associated mechanical allodynia.	[142]
	Aggrecan turnover is Adamts5-mediated, and lack of Adamts5 results in adverse effects on structure and function of tendon and enthesis. FDL tendon (a single fascicle lacking prominent insertion site) of 12-week-old *Adamts5* ^−/−^ mice showed structural abnormalities and decreased material properties as a result of accumulation of aggrecan in the pericellular matrix of tendon fibroblasts. In Achilles tendon (a multifascicles tendon with defined insertion of tendon fibers), *Adamts5* ^−/−^ mice exhibited higher tensile modulus and weak enthesis in mutants.	N/A	[143]

*Prg4* ^ tm1Mawa^: proteoglycan 4; targeted mutation 1, Matthew Warman Allele type: T (KO) Mut: insertion (IGD) Syn: *Lubricin* ^−^	Lack of Prg4 causes abnormal calcification of tendon and sheath involved in ankle joints leading to precocious joint failure. The morphologic changes in 7-month-old *Prg4* ^−/−^ mice was compared to *Prg4* ^+/−^ mice. Homozygous mutants demonstrated abnormal calcification of tendon sheaths of tibialis anterior that surrounds the ankle joint. Absence of Prg4 within the tendon sheath results in decreased lubrication leading to tissue damage and dystrophic calcification. These pathological changes within tendon and tendon sheath in *Prg4* ^−/−^ mice resulted in camptodactyly similar to that observed in human CACP patients.	*Prg4* ^−/−^ mouse was generated. Aging *Prg4* ^−/−^ mice show progressive joint failure. *Prg4* ^−/−^ mouse is a model for human CACP; OMIM 208250.	[144]
	Prg4 plays a role in interfascicular lubrication in tendon. The study involved pulling of a 15 mm long fascicle segment proximally from a distally cut tail of 10–16-week *Prg4* ^−/−^, *Prg4* ^+/−^, and *Prg4* ^+/+^ mice. The mean peak gliding resistance was more in homozygote mutant than Het and WT mice (43.2, 35.4, and 28.5 mN, resp.).	N/A	[145]
	Prg4 expression plays a role in the viscoelastic properties of tail tendon fascicles. Tendon stiffness and viscoelasticity was determined in 10–13-week *Prg4* ^−/−^, *Prg4* ^+/−^, and *Prg4* ^+/+^ mice. A ramp test was used to determine the elastic modulus by pulling the fascicles to 2.5% strain amplitude at a rate of 0.05 mm/s followed by a relaxation test that pulled the fascicles to 5% strain amplitude at a rate of 2 mm/s. The fascicles were allowed to relax for 2 min at the maximum strain and a single-cycle relaxation ratio was used to characterize the viscoelastic properties. *Prg4* homozygous mutant mice had lower relaxation ratio than the WT mice.	N/A	[146]

Congenital camptodactyly (Human case report)	In two sisters with congenital camptodactyly and joint effusions, abnormalities in tendons were restricted to the portion within synovial sheaths implying a disease of the tenosynovium. In areas of chronic involvement, some tendons were replaced by fibrous tissue. Fingers, in the patient with the disease, had hard scarring tissue.	N/A	[147]

*PRG4* (*Homo sapiens*) Syn: CACP, lubricin	Mutations in *CACP/PRG4* gene, causing disease, were identified as four deletion mutations (2805del5, 3240del7, 3023del2, and 3690del5) that alter the reading frame and result in premature truncation of the full-length polypeptide.	Human disease: CACP; OMIM: 208250.	[148]

*PRG4* (*Homo sapiens*)	Sequence analysis of *PRG4* gene, in the affected individuals of a Pakistani family with CACP syndrome, revealed a 2-base-pair deletion (c.2816_2817delAA) predicting a frame shift mutation (p.Lys939fsX38).	Human disease: CACP OMIM: 208250.	[149]

## Abbreviations

BMP: Bone morphogenetic protein
BMPR2: Bone morphogenetic protein receptor, type II
Col3a1: Collagen, type III, alpha 1
CACP: Camptodactyly-arthropathy coxa vara-pericarditis syndrome
Dbl-KO: Double knockout
E: Embryonic day
ECM: Extracellular matrix
EDS: Ehlers-Danlos syndrome
FDL: Flexor digitorum longus tendon
GAG: Glycosaminoglycan
IHC: Immunohistochemistry
Insertion (IGD): Insertion, intragenic deletion
ISH: In situ hybridization
Loc: Location
LRR: Leucine-rich repeat
LRRNT: LRR containing N-terminal domain
Mut: Mutation
OA: Osteoarthritis
P: Postnatal day
PDGFb: Platelet-derived growth factor *β* polypeptide chain
PDL: Periodontal ligament
SEM: Scanning electron microscopy
SLRP: Small leucine-rich proteoglycan
Syn: Synonym
T (KO): Targeted (knockout)
T (KI): Targeted (knockin)
T (R): Targeted (reporter)
TEM: Transmission electron microscopy
Tgf*β*: Transforming growth factor beta
tm1: Targeted mutation 1
WT: Wild type (control mouse)
N/A: Phenotype not available, that is, phenotype not studied by the authors in that reference paper.
